# GRACE: Generative Redesign in Artificial Computational Enzymology

**DOI:** 10.1021/acssynbio.4c00624

**Published:** 2024-11-08

**Authors:** Ruei-En Hu, Chi-Hua Yu, I-Son Ng

**Affiliations:** † Department of Chemical Engineering, National Cheng Kung University, Tainan City 701, Taiwan; ‡ Department of Engineering Science, National Cheng Kung University, Tainan City 701, Taiwan

**Keywords:** *de novo* enzyme, deep learning, protein solubility, protein classification, molecular docking

## Abstract

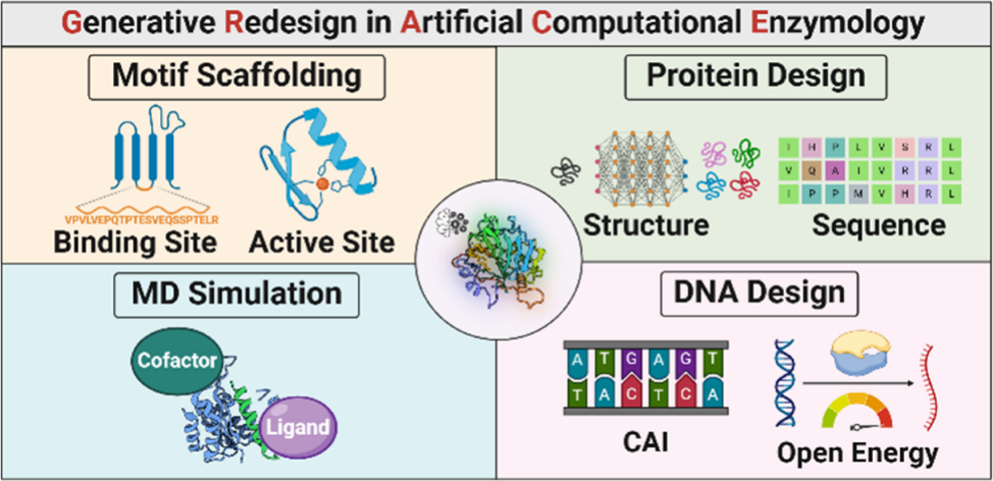

Designing *de novo* enzymes is complex and challenging, especially to maintain the activity. This research focused on motif design to identify the crucial domain in the enzyme and uncovered the protein structure by molecular docking. Therefore, we developed a Generative Redesign in Artificial Computational Enzymology (GRACE), which is an automated workflow for reformation and creation of the *de novo* enzymes for the first time. GRACE integrated RFdiffusion for structure generation, ProteinMPNN for sequence interpretation, CLEAN for enzyme classification, and followed by solubility analysis and molecular dynamic simulation. As a result, we selected two gene sequences associated with carbonic anhydrase from among 10,000 protein candidates. Experimental validation confirmed that these two novel enzymes, *i.e.*, dCA12_2 and dCA23_1, exhibited favorable solubility, promising substrate-active site interactions, and achieved activity of 400 WAU/mL. This workflow has the potential to greatly streamline experimental efforts in enzyme engineering and unlock new avenues for rational protein design.

## Introduction

Enzymes, as specialized proteins, play a crucial role in biological systems by acting as catalysts that facilitate a wide range of essential chemical reactions required for life.^1^ Despite their natural efficiency, enzymes often face critical limitations when applied in industrial and therapeutic demand. The limitations include poor enzymatic stability, narrow substrate specificity, and low tolerance to solvent, which restrict the broader utility.^2,3^ To overcome such challenges, enzyme engineering has emerged by focusing on the enhancement and modification of enzyme properties to better suit specific applications across biotechnology, medicine, and environmental science.^4^

By tailoring the enzyme structure and function to operate under diverse conditions, protein engineering offers the potential to revolutionize multiple fields. This includes enabling more sustainable chemical processes, the development of innovative therapeutic solutions, and the creation of advanced approaches to address global challenges such as energy production and environmental remediation.^5,6^ As this field continues to evolve, it promises to unlock new possibilities for utilizing enzymes in ways that are previously unattainable, significantly advancing both scientific understanding and practical applications.^7^

Carbonic anhydrase (CA; EC 4.2.1.1) represents one of the most ancient enzyme families on Earth, with seven distinct classes exhibiting diverse evolutionary patterns. This ubiquitous enzyme, found across all kingdoms of life, catalyzes the interconversion of carbon dioxide and bicarbonate, and has emerged as a valuable biomarker for various diseases.^8^ Human carbonic anhydrase II (hCAII) is an exceptionally efficient enzyme among natural CAs, largely due to its unique active site structure, which is crucial for interconversion of carbon dioxide and bicarbonate.^9^ However, natural CAs often suffer from limited thermostability and low protein expression yields when obtained from natural sources. Thus, there is an urgent need to design enzymes with enhanced stability, improved activity, or easier expression.^10^

The creation of *de novo* enzymes with specific functions has become pivotal for advancing biotechnology. Traditional methods, which rely on random mutagenesis and extensive screening,^11^ are often hampered by inefficiencies and a limited understanding of the relationship between protein sequence and function.^12^ In contrast, the inverse design addresses such challenges by first defining the desired protein function and then identifying the corresponding sequences. This approach efficiently navigates the sequence space, enabling the production of proteins with precise specifications and allowing for the systematic design and optimization. Therefore, a targeted strategy is essential for driving innovative solutions to some of the most critical global challenges.

Recently, deep learning-based models for enzyme engineering have emerged as powerful tools for predicting enzyme properties and designing novel sequences.^13,14^ All models can learn complex patterns from large data sets of enzyme sequences and structures, capturing subtle relationships that traditional methods may overlook. By integrating the predictive power of deep learning with conventional approaches, more efficient and effective enzyme engineering strategies have been developed.^15^

Alongside the rapid advancement of machine learning and deep learning models, some groundbreaking tools have been developed to overcome key challenges in structural biology. Notably, AlphaFold^16^ and RoseTTAFold^17^ have demonstrated remarkable precision in predicting protein structures from sequence information, while ProteinMPNN has facilitated the reverse engineering of protein sequences by leveraging structural data.^18^ Additionally, models such as RFdiffusion^19^ and EvoDiff^20^ have shown great promise in generating new protein structures or sequences. Researchers have successfully employed a family wide hallucination approach to generate *de novo* luciferase enzymes with higher substrate specificity and activity and have achieved record-high affinity in protein binder complexes.^21^ All of the strategies have led to significant advancements in the evolution of enzymes and peptides. However, current methods for validating *de novo* enzymes still rely heavily on large-scale and high-throughput experiments. A systematic approach to generating enzymes with specific functions, combined with an *in silico* rational screening platform, is crucial but remains under development.

In this study, we focused on hCAII to identify key motifs contributing to its enzymatic activity and further design structures that retain the enzyme’s original functionality. We developed a comprehensive workflow that incorporated the generation and *in silico* screening of *de novo* enzyme designs. The resulting sequences were evaluated by using molecular dynamics (MD) simulations, which predicted promising enzyme–substrate interactions. Finally, the candidate enzymes were synthesized and validated. Overall, we provided the streamline to create novel enzymes from GRACE, a Generative Redesign using Artificial Computational Enzymology.

## Results and Discussion

### Motif Recognizing and Scaffolding on hCAII

**Figure 1:**
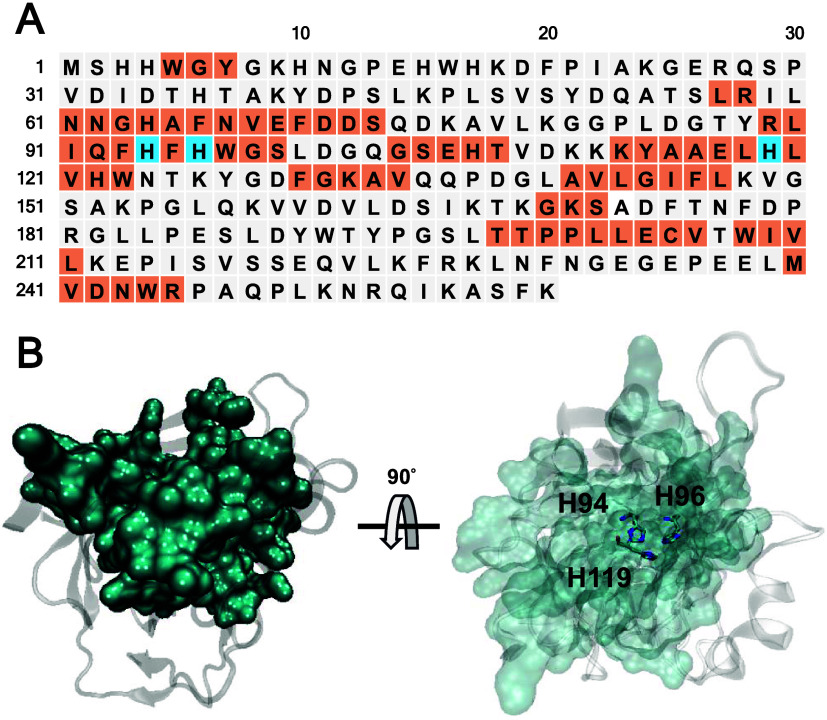
Motif recognition and selection in human carbonic anhydrase (hCAII). (A) The protein sequence of hCAII is shown, with the selected motifs highlighted in orange, while the catalytic triad, consisting of H94, H96, and H119, is marked in blue color. (B) The 3D structure of hCAII is displayed, where the selected motifs (in cyan) form a deep cavity, with the catalytic triad (H94, H96, and H119) positioned at the bottom of the cavity.

The active site of hCAII is located within a deep, cone-shaped cavity where a catalytically essential zinc ion (Zn^2+^) is coordinated by three histidine residues: H94, H96, and H119 (Figure 1A).^9^ The binding site of hCAII extends beyond the catalytic zinc ion and the catalytic triad, involving a network of residues that facilitates substrate binding, catalysis, and product release. Key residues such as T199 and E106 play vital roles in orienting the substrate (HCO_3_^–^) and stabilizing reaction intermediates,^22^ while the hydrophobic pocket responsible for CO_2_ binding is formed by residues like V121 and V143, creating an environment conducive to substrate interaction.^23^ Moreover, H64 functions as a critical proton shuttle during the catalytic process, and N62 is involved in the hydrogen-bonding network within the active site.^24^ On the other hand, residue of W209 helps maintaining the structural active site, while F131 contributes to both the hydrophobic pocket and substrate specificity.^25^ The critical binding sites and cavity-forming residues (Figure 1B) were therefore selected as motifs for the *de novo* generation of carbonic anhydrase in the following.

### Automated Design Workflow for *De Novo* Enzymes

**Figure 2:**
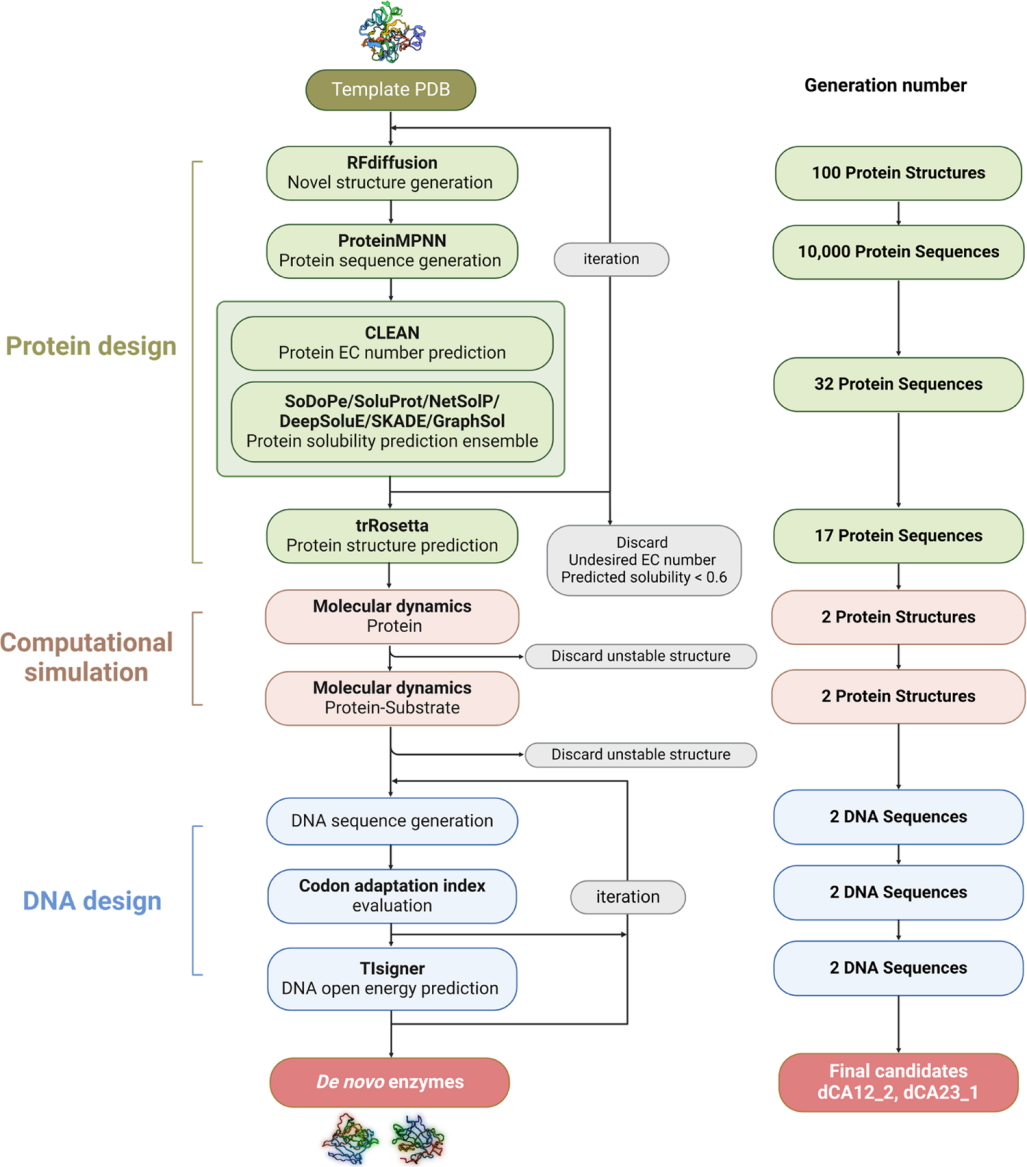
Automated workflow for *de novo* enzyme design. In translational design, novel backbone structures are generated using RFdiffusion, followed by interpretation *via* ProteinMPNN. Candidate enzymes are screened by CLEAN which predicts the EC number, and their solubility is accessed using a solubility ensemble model. The predicted structures generated by trRosetta are further evaluated for stability and activity with target substrate through molecular dynamic (MD) simulations. Finally, in transcriptional design, DNA sequences for the enzyme candidates are generated and optimized using codon adaptation index and DNA opening energy, facilitated by TIsigner (https://tisigner.com).

First, we developed an automated workflow that integrates three modules, protein design, computational analysis, and DNA design, to obtain the novel enzymes (Figure 2). In the protein design module, new backbone structures were generated from a given template structure sourced from the protein data bank (PDB) using RFdiffusion. Next, protein sequences were predicted based on ProteinMPNN and confirmed the enzymatic classification (*i.e.*, EC number) using CLEAN.^26^ Sequences that matched the correct EC number (*i.e.*, 32 sequences passed) and had high solubility probabilities (>0.6) were further analyzed using trRosetta,^27^ ensuring the selected proteins had well-defined structures.

In the computational module, the focus is to discard the unstable structure through molecular dynamics analysis of protein and its structure. In this case, only 2 candidate structures remained to the DNA design module, in which the corresponding DNA sequences were decoded from the protein sequences. We used the Codon Adaptation Index (CAI) (https://www.biologicscorp.com/tools/CAICalculator/) to ensure CAI value >0.6 for positive expression.^28^ Additionally, the first 30 nucleotides of the sequences were optimized using TISigner^29^ to reducing the opening energy of target sequence, which is successfully to improve recombinant protein expression recently.^30^

### Evaluation of *De Novo* Enzymes under Structure and Sequence Generator

To determine the most suitable generation model, we evaluated several state-of-the-art models, including sequence generation models of Progen2,^31^ EvoDiff, and DLPM,^32^ as well as structure generation model of RFdiffusion coupled with inverse folding models (*i.e.*, ProteinMPNN and CarbonDesign).^33^ For fair comparison of all models, unconditional sequence generation was used at first and the quality,, diversity, and novelty was further analyzed by ProteinBench^34^ in terms of benchmark metrics, such as self-consistent TM score (scTM), predicted local distance difference test (pLDDT), pairwise TM score (pairwise TM), max clustering ratio (Max Clust.), and max TM score to PDB database (Max TM).

As shown in Table 1, Progen2, EvoDiff, and DPLM showed large differences in the quality, diversity, and novelty. Progen2 showed balanced performance across the three metrics compared to EvoDiff and DPLM, with quality increasing along with larger parameter sizes. However, the extremely high pairwise TM and low max clustering ratio in Progen2-large indicated that it generated almost identical enzyme sequences. Thus, larger parameter size in Progen2 is unnecessary, whereas Progen2-base showed greater potential in sequence generation due to its balancing performance. EvoDiff demonstrated strength in structure diversity and novelty with the lowest pairwise TM, lowest Max TM, and highest max clustering ratio. However, the quality of EvoDiff’s generated sequences was low, with pLDDT scores (<0.4). DPLM had the highest quality within sequence generation models, but the generated sequences were similar to each other, which DPLM achieved the highest Max TM. To sum up, Progen2, EvoDiff, and DPLM each have their pros and cons, and the choice among them should be task oriented.

**Table 1:** Sequence Performance of Protein Sequence Generative Models, Progen2, EvoDiff, and DPLM^a^

	evaluation metric			
	quality	diversity		novelty
model type	pLDDT	pairwise TM	max clust.	max TM
Progen2-small	47.33	0.50	0.34	0.55
Progen2-base	48.51	0.63	0.40	0.54
Progen2-large	72.14	0.95	0.08	0.84
EvoDiff-oadm_38 M	31.58	0.43	0.89	0.52
EvoDiff-oadm_640 M	32.19	0.42	0.84	0.55
EvoDiff-d3pm_uniform_38 M	26.95	0.43	0.85	0.49
EvoDiff-d3pm_uniform_640 M	26.84	0.41	0.85	0.45
EvoDiff-d3pm_blosum_38 M	28.75	0.47	0.84	0.52
EvoDiff-d3pm_blosum_640 M	30.32	0.50	0.82	0.53
DPLM _150 M	85.59	0.77	0.41	0.90
DPLM _650 M	83.47	0.73	0.55	0.88

a pLDDT (Predicted local distance difference test) measures the per-residue local confidence with an experimental structure. pLDDT ranges from 0 to 100. Higher pLDDT means more accurate and considered to be stabler structure.

For the inverse folding models of ProteinMPNN and CarbonDesign, the metrics scTM and pLDDT were used to measure the refoldability and stability of the generated sequences. As shown in Table 2, the overall performance of ProteinMPNN and CarbonDesign was similar at all sampling temperatures. Increased sampling temperature decreased refoldability and stability. However, the slightly higher scTM of ProteinMPNN at a sampling temperature 0.8 showed greater noise durability compared to CarbonDesign, where noise is a factor to improve generation quality in inverse folding models.^35^

**Table 2:** Structural Performance of *De Novo* Backbones and Based Sequence Design Using Inverse Folding Models ProteinMPNN and CarbonDesign^a^

		evaluation metric	
		refoldability	stability
model	sampling temperature	scTM	pLDDT
proteinMPNN	0.1	0.99	60.42
	0.2	0.99	61.21
	0.5	0.94	47.81
	0.8	0.76	34.80
	1.0	0.48	29.86
	1.2	0.46	26.30
	1.5	0.42	24.38
	2.0	0.37	22.65
CarbonDesign	0.1	0.99	59.94
	0.2	0.99	61.79
	0.5	0.99	50.56
	0.8	0.60	37.69
	1.0	0.53	32.27
	1.2	0.46	28.71
	1.5	0.43	25.00
	2.0	0.38	22.80

a The structures were predicted using ESMFold for accelerated prediction speed compared with AlphaFold 2. scTM (self-consistent template modeling score) measures the consistency between mother template structure and designed structure from inverse folding models. scTM ranges from 0 to 1. Scores higher than 0.5 are considered to be refoldable and self-consistent.

**Figure 3:**
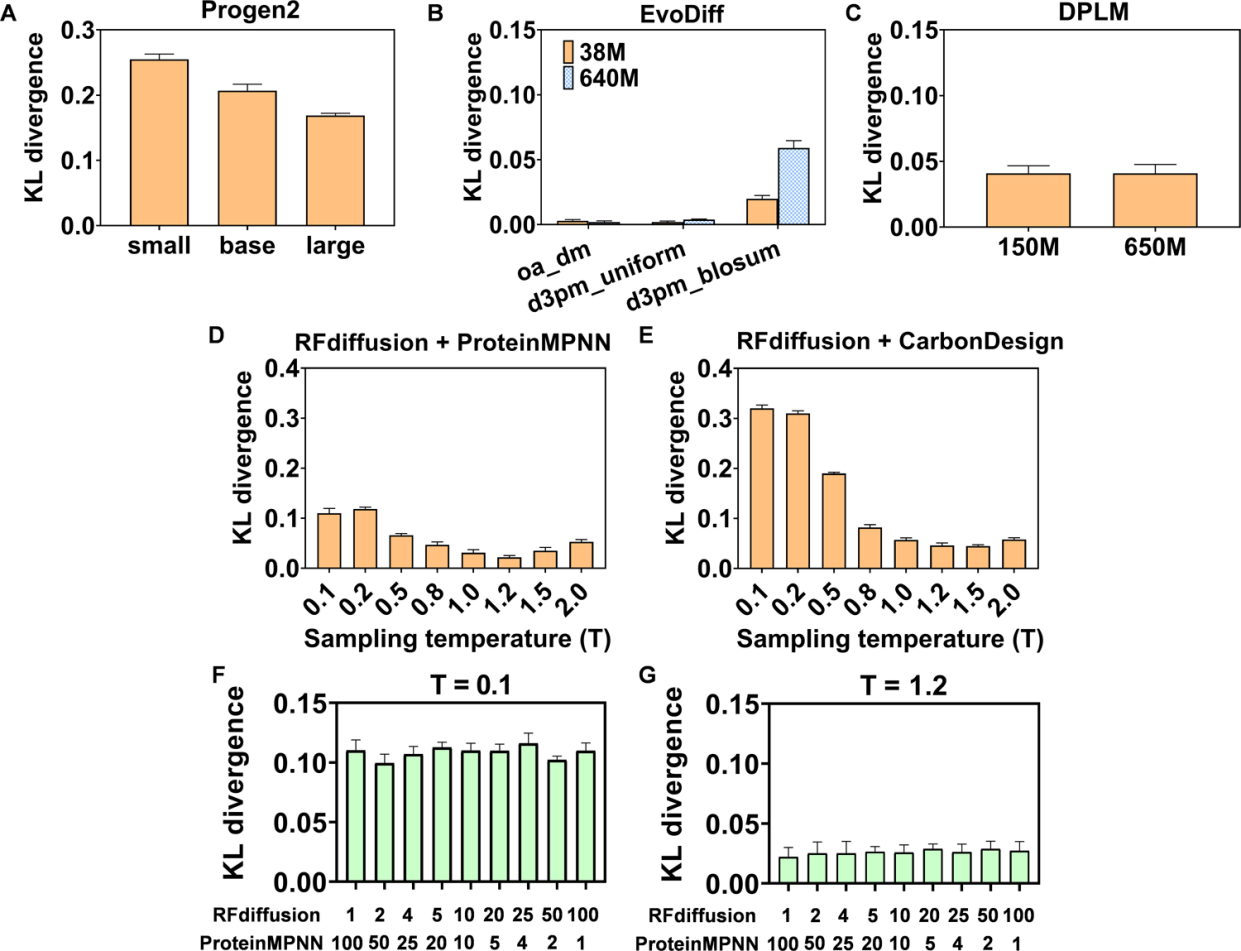
KL divergence of 100 unconditional generated sequences from different sequence generation models (*i.e.*, Progen2, EvoDiff, and DPLM) and structure generation models coupled with inverse folding models (*i.e.*, RFdiffusion + ProteinMPNN and RFdiffusion + CarbonDesign) at several sampling temperatures. (A) Progen2: The models progen2-small, progen2-base, and progen2-large are used, varying by parameters size (151 M for progen2-small, 764 M for progen2-base, 2.7B for progen2-large). (B) EvoDiff: The models used are oa_dm_38 M to 640M, d3pm_blosum_38 M to 640M, d3pm_uniform_38 M to 640M, differentiated by the training algorithm (order-agnostic autoregressive diffusion for “oa”; discrete denoising diffusion probabilistic models for “d3pm”; uniformly sampled amino acids for “uniform”; BLOcks SUbstitution Matrix for “blosum”) and parameters size (38 M or 640M). (C) DPLM: The models are including dplm_150 M and 650M, respectively. (D) RFdiffusion + ProteinMPNN: KL divergence is evaluated using sampling temperatures from *T* = 0.1 to 2.0, with the ratio of RFdiffusion to ProteinMPNN fixed at 1:100. (E) RFdiffusion + CarbonDesign: Similar sampling temperatures are applied as in the RFdiffusion + ProteinMPNN setup, with the ratio of RFdiffusion to CarbonDesign also set at 1:100. Panels (F, G) show the KL divergence of 100 unconditionally generated sequences from RFdiffusion + ProteinMPNN at specific sampling temperatures (0.1 in F and 1.2 in G), under different sequence/structure combinations.

In addition to the general metrics in ProteinBench, we also measured Kullback–Leibler (KL) divergence using the Uniref50 database to assess the quality of the generated sequences from all models (Figure 3). The amino acid composition differed significantly from natural proteins for Progen2 (Figure S1A), resulting in the highest KL divergence among the models (Figure 3A,E). This high divergence suggested that Progen2 has limitations for *de novo* enzyme generation. For instance, more than 500 sequences could be generated in a batch using the progen2-small model, while the amounts drastically dropped to 80 for the progen2-base model, and fewer than 10 sequences were generated in a batch with progen2-large. The combination of low intrinsic performance and high computational demands made Progen2 unsuitable for high-throughput *de novo* enzyme sequence generation. In contrast, EvoDiff showed the lowest KL divergence among the three models (Figure 3B); its amino acid composition was closely aligned with natural enzymes (Figure S1B). For DPLM, the KL divergence showed a moderate value (Figure 3C), and the amino acid composition was also close to that of natural enzyme sequences (Figure S1C). The low KL divergence of EvoDiff and DPLM exhibited potential for generating rational sequences.

The KL divergence was moderate in RFdiffusion with ProteinMPNN (Figure 3D). Additionally, ProteinMPNN allowed the adjustment of the sampling temperature, which affected the diversity of sequences and their amino acid content (Figure S2A), demonstrating its flexibility. When considered RFdiffusion with CarbonDesign, the KL divergence was much higher than that of ProteinMPNN-generated sequences. Furthermore, the amino acid content was slightly different from natural enzymes (Figure S2B). The lower divergence and better amino acid content of ProteinMPNN further demonstrated its good performance. Interestingly, higher sampling temperatures led to more amino acid generation frequencies in both ProteinMPNN and CarbonDesign (Figure S2).

To assess the impact of the ratios on structure and sequence, we tested different ratios of RFdiffusion and ProteinMPNN for 100 unconditional sequences (Figure 3F,G). The results showed that the sampling ratio did not compromise generation quality (Figures S3 and S4). Adjusting the sampling temperature not only influenced sequence diversity but also affected ProteinMPNN’s codon preferences,^18^ offering greater flexibility in enzyme design. Consequently, the better inverse folding model relied on ProteinMPNN, RFdiffusion, and ProteinMPNN in our workflow.

### Get What You Came for: *De Novo* Enzyme Screening via EC Number

Typically, a high-throughput screening platform is employed after *de novo* enzyme designs to select the sequences with the highest expression and authentic activity.^36^ However, this approach is labor-intensive and may not always yield positive results due to challenges such as protein expression issues, poor solubility, or even loss of activity due to unwanted motifs. Thus, establishing a robust criterion for screening potential candidates from the pool of generated sequences is essential.

**Figure 4:**
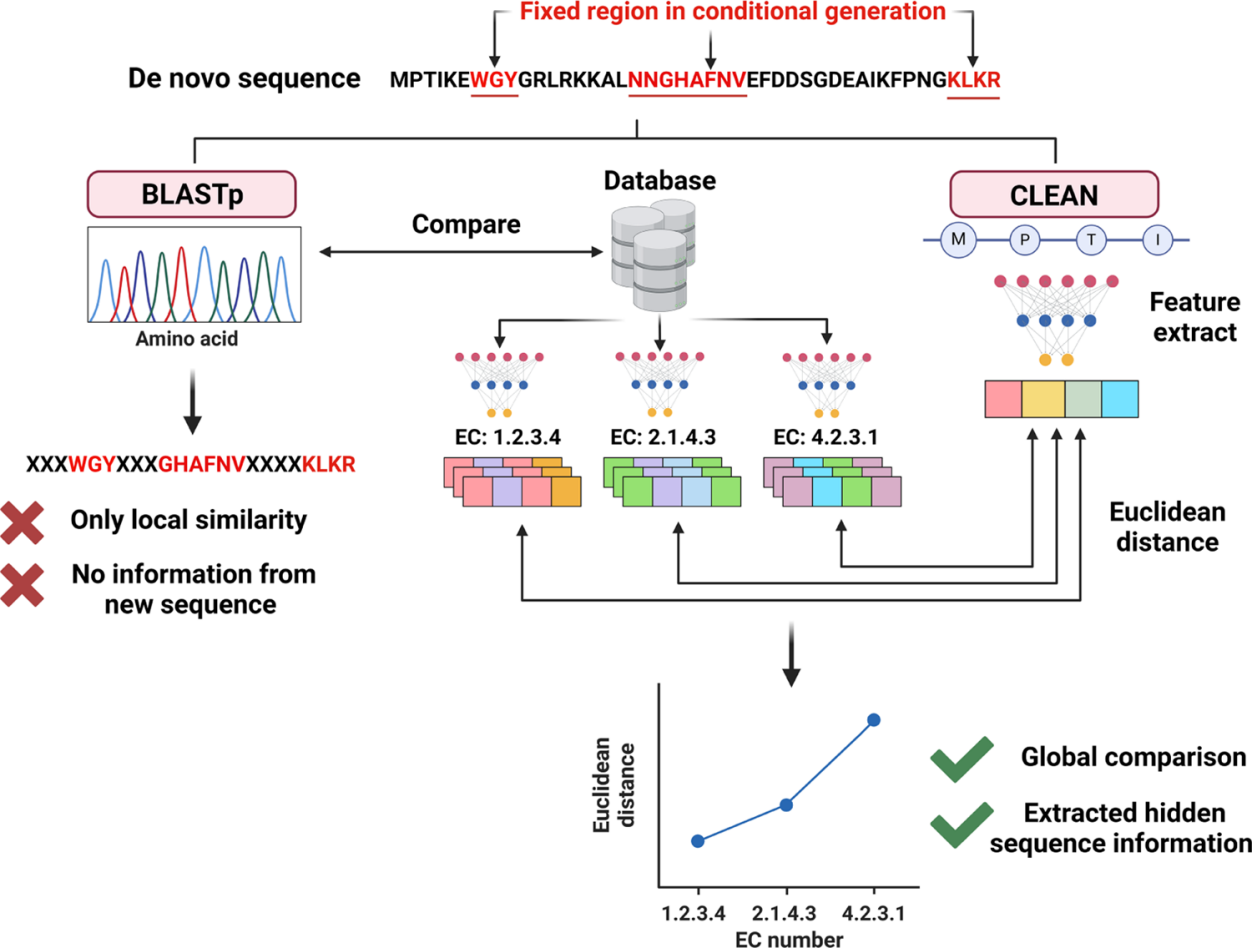
Comparison of BLASTp and CLEAN in sequence classification for *de novo* enzymes. BLASTp performs local sequence alignment, providing only local similarity information without capturing the full context of the new sequence. In contrast, CLEAN utilizes a feature extraction process to analyze the entire sequence, enabling global comparison and extraction of hidden sequence information. The *de novo* sequence is compared against a database, in which CLEAN is capable of distinguishing between different EC numbers through Euclidean distance calculations based on extracted features.

BLASTp effectively predicts homologous proteins by searching for sequence similarities. Due to differences between *de novo* protein sequences and existing databases, it only recognizes conserved regions manually specified during the initial backbone structure design with RFdiffusion (Figure 4). Therefore, CLEAN,^26^ which utilizes contrastive learning based on sequence information, can predict the specific EC number of the target enzyme with remarkable accuracy compared to existing models and BLASTp. CLEAN’s ability to extract sequence patterns allows it to prevent the incorporation of undesired motifs from other enzymes, ensuring the design remains specific to the desired enzyme family.

### Solubility Matters: *De Novo* Enzyme Screening via Solubility Ensembles

Protein expression and solubility pose significant challenges for experimental validation, particularly when working with proteins not naturally occurring.^37^ Several predictive models have been developed to address solubility based on protein sequences, such as DeepSoluE and SoluProt.^38,39^ Some models, like NetSolP,^40^ utilize pretrained protein language models to improve performance, while others, like SKADE,^41^ SoDoPe,^42^ and GraphSol,^43^ are based on protein structural information. Specifically, GraphSol integrates multiple protein feature maps, including amino acid encoding *via* Blosum62, physicochemical properties, evolutionary conservation, and predicted structural features, leading to a high prediction accuracy. Unlike models that provide binary predictions of solubility, GraphSol predicts the proportion of the soluble protein within the total protein.

When solubility predictions for hCAII were compared, discrepancies were observed across different models, with some predictions deviating significantly from actual solubility (Table 3 and Figure S5). For example, SKADE predicted hCAII as likely insoluble, contradicting experimental results, which led to its exclusion from our design workflow. In contrast, SoluProt and DeepSoluE predicted hCAII to be soluble with high confidence. Notably, GraphSol demonstrated potential for experimental protein screening, although it slightly underestimated hCAII’s soluble ratio.

**Table 3:** Real and Predicted Solubility Score of hCAII and Two *De Novo* Enzymes Using SoDoPe, SoluPort, NetSolP, DeepSoluE, SKADE, and GraphSol^a^

		soluble probability					soluble ratio
enzyme	real soluble ratio	SoDoPe	SoluProt	NetSolP	DeepSoluE	SKADE	GraphSol
hCAII	0.88	0.76	0.93	0.68	0.99	0.59	0.63
dCA12_2	0.40	0.87	0.60	0.51	0.66	0.63	0.72
dCA23_1	0.23	0.82	0.68	0.52	0.75	0.56	0.67

a The soluble ratio from real experimental data and GraphSol represent the ratio of soluble protein to the whole cell protein.

Different solubility models may interpret protein characteristics and solubility in varied ways. To mitigate the bias of relying on a single model, we incorporated three solubility models, SoDoPE, SoluProt, and GraphSol, into our workflow. The *de novo* designed enzymes dCA12_2 and dCA23_1 exhibited solubility probabilities and soluble ratios exceeding 0.6, marking them as promising candidates for further study.

### Fierce Competition on Precise Structure Prediction of *De Novo* Proteins

Previously, protein structure determination using cryo-electron microscopy or tomography has been a reliable approach.^44^ However, these methods present the labor-intensive nature of working with a large number of existing proteins or the limited availability of data for less-studied proteins. As a result, there has been a growing demand for software-based structure prediction in recent years. Deep learning-based methods have achieved the highest accuracy, gaining prominence since AlphaFold 2′s breakthrough performance in the 2020 CASP14 (Critical Assessment of Protein Structure Prediction) competition. Since then, several protein structure prediction models with various strategies have evolved, such as AlphaFold 3^45^ and trRosetta, the present newest model from Google DeepMind and top ranked server of CASP15, respectively. Herein, the structural assessment of *de novo* enzymes was tested by using AlphaFold 2, AlphaFold 3, and trRosetta. Model’s performance was evaluated using the predicted template modeling (pTM) score, representing the confidence level in the overall predicted structure, and predicted local distance difference test (pLDDT), measuring the per-residue local confidence with an experimental structure.

As shown in Table 4, trRosetta demonstrated high accuracy for the enzymes dCA12_2 and dCA23_1, with pTM scores of 0.85 and 0.81, respectively. AlphaFold 2 showed the relatively lowest pTM scores of 0.68 and 0.63 compared with the other models. While AlphaFold 3 showed a slightly higher pTM score to 0.70 and 0.63 compared with AlphaFold 2, the pTM scores were not higher than 80 in all of the *de novo* enzymes. Additionally, trRosetta had a slightly higher pLDDT score compared to the other models. Indeed, the accuracy score cannot exceed 80 when shallow MSA depth is provided in AlphaFold 3, in which the *de novo* enzymes might have difficulty in getting precise structure.^45^

**Table 4:** Prediction Confidence Score from Models AlphaFold 2, AlphaFold 3, and trRosetta

	AlphaFold 2		AlphaFold 3		trRosetta	
enzyme	pTM	pLDDT	pTM	pLDDT	pTM	pLDDT
dCA12_2	0.68	62	0.70	65	0.85	68
dCA23_1	0.63	58	0.63	61	0.81	61

Figure S6 displays the structural predictions of enzymes, focusing on their tertiary structures and catalytic triads, which are critical for enzymatic activity. dCA12_2, consisting of 219 amino acid residues, featured a catalytic triad formed by H68, H70, and H112 (Figure S6A). dCA23_1, with 181 amino acids, had a catalytic triad formed by H48, H50, and H75 (Figure S6B). The arrangement of catalytic triads in dCA12_2 and dCA23_1 is similar to that of natural enzyme hCAII, but both *de novo* enzymes have shorter residue lengths.

### *In Silico* Validation of dCA Using Molecular Dynamics and Enzymatic Activity

As shown in Figure S7A, only neighboring residues of the critical active sites were selected as motifs, excluding the fixed region with essential 3 β-sheets and cavity-forming residues (Figure S7B). Experimental validation revealed a drop in CA to 50 WAU/mL (Figure S7C), indicating the importance of cavity-forming residues of hCAII in maintaining the activity. Furthermore, MD simulations can help identify low-performing candidates, reducing the need for labor-intensive wet experiments.

**Figure 5:**
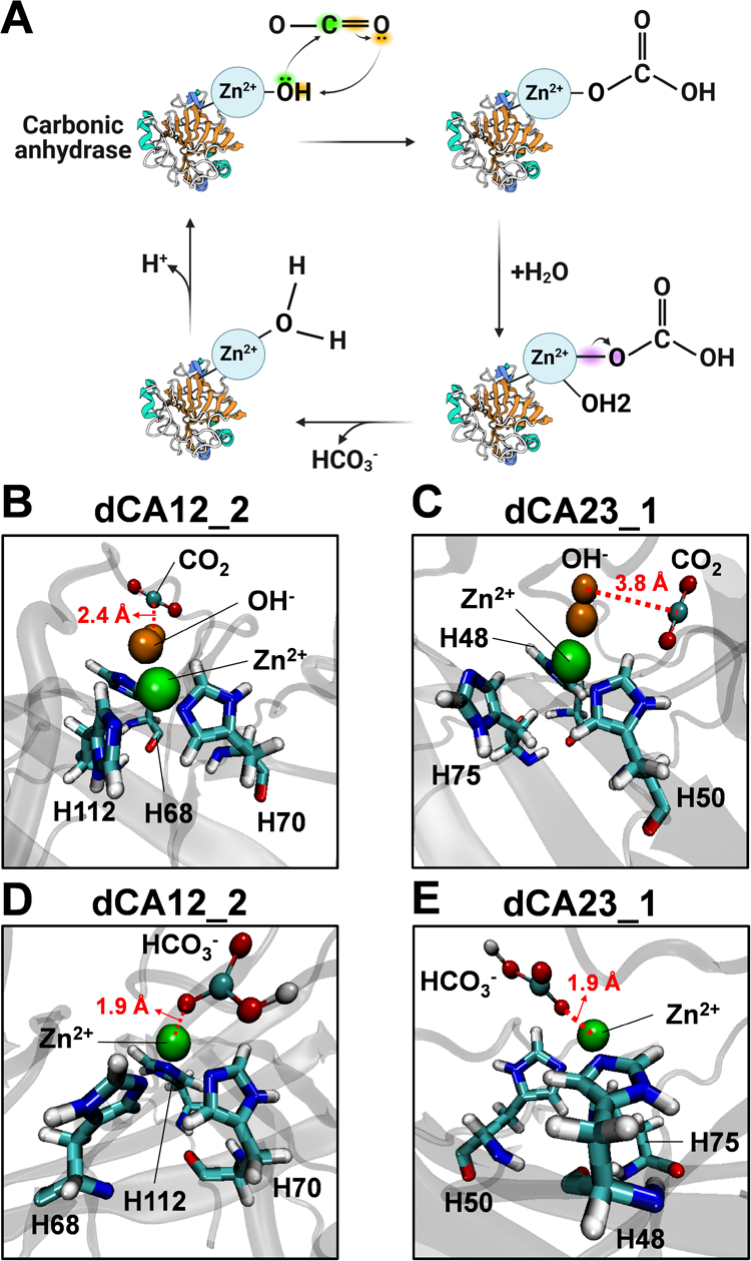
Reaction mechanism and molecular docking of carbonic anhydrase. (A) The catalytic mechanism of carbonic anhydrase, illustrating the binding of CO_2_ to the zinc-bonded OH^–^ at the active site of (B) dCA12_2 and (C) dCA23_1, respectively. Binding of ligand HCO_3_^–^ with the zinc ion at the active site of (D) dCA12_2 and (E) dCA23_1.

The catalytic mechanism of carbonic anhydrase is depicted in Figure 5A. The zinc ion cofactor initially binds to the active site triad, which is primarily composed of three histidine residues. In this mechanism, the zinc-bound hydroxide ion (Zn^2+^–OH^–^) attacks the carbon atom of an incoming carbon dioxide molecule (CO_2_), leading to the formation of bicarbonate (HCO_3_^–^) during a nucleophilic attack. Subsequently, a water molecule binds to the reaction intermediate, facilitating an internal proton transfer results in the release of bicarbonate. Finally, the zinc-bound water undergoes deprotonation, regenerating Zn^2+^–OH^–^ and completing the catalytic cycle.^46^

To validate the potential catalytic activity of dCAs, MD simulations were conducted, which focused on interactions between the dCAs, zinc ion, hydroxide ion, and substrates CO_2_ or HCO_3_^–^. The simulations were divided into two schemes to observe different binding profiles: Zn^2+^–OH^–^ with CO_2_ and Zn^2+^ with HCO_3_^–^. Both dCA12_2 and dCA23_1 showed correct coordination of the zinc ion within the three-histidine active site with the hydroxide ion positioned near the zinc ion. The ligand CO_2_ was also observed to be in close proximity to the zinc-bound hydroxide, with distances of 2.4 and 3.8 Å, respectively (Figure 5B,C). Additionally, the bicarbonate ion (HCO_3_^–^) was positioned near the active site (Figure 5D,E), interacting with the catalytic residues and the zinc ion. The distances between the zinc ion and the oxygen atoms of HCO_3_^–^ in both dCA12_2 and dCA23_1 were measured at 1.9 Å, indicating potential ion-binding interactions that are crucial for the catalytic mechanism.

**Figure 6:**
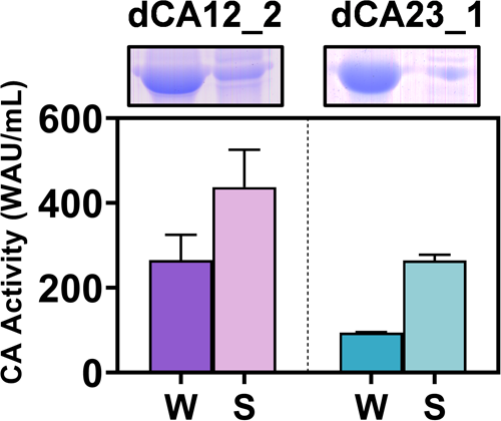
Enzymatic characterization and protein expression of dCA12_2 and dCA23_1. The CA activity (bottom) and the corresponding protein expression (top) of dCA12_2 and dCA23_1, while W and S represent whole cell and soluble crude protein, respectively.

As shown in Figure 6, dCA12_2 and dCA23_1 exhibited successful protein expression, while CA activity was 400 and 260 WAU/mL, respectively. The relatively higher activity of dCA12_2 corresponded to the MD simulation in which CO_2_ had a closer distance with the catalytic triad of dCA12_2 to 2.4 Å, whereas the relatively low activity of dCA23_1 may result from the weak interaction between CO_2_ and the catalytic triad.

The *de novo* enzyme design of sequentially and structurally diverse and smaller enzymes have a high industrial and pharmaceutical value.^47^ In industrial biocatalysis, our workflow could improve the chemical process efficiency and enable novel bioprocesses. In pharmaceuticals, the automated motif scaffolding accelerates therapeutic enzyme development, facilitating complex synthesis, reducing costs, and advancing targeted drug delivery systems.^48^

## Conclusions

When hCAII was used as the template, we demonstrated our workflow successfully generated 2 *de novo* enzymes, dCA12_2 and dCA23_1, which have true activity and expression. The current result is tested in designing monomer proteins; thus, designing more complex proteins or multimers is still a challenge. On the other hand, incorporating enzyme activity prediction model such as DLKcat^49^ may further improve the robustness of our workflow. The portraits of this workflow set up the benchmark on effective *de novo* enzyme design, broaden the structure or sequence space of the natural enzymes, and even accelerate the development of new enzymes.

## Materials and Methods

### Progen2, EvoDiff, DPLM, RFdiffusion with ProteinMPNN or CarbonDesign for Enzyme Generation

Progen2, EvoDiff, DPLM, RFdiffusion, ProteinMPNN, and CarbonDesign were used as models for evaluating *de novo* protein generation. For RFdiffusion, ProteinMPNN or CarbonDesign served as the protein sequence decoder, as RFdiffusion generates only the protein backbone. For evaluating the *de novo* protein generators, the number of sequences generated per input structure was adjusted based on different combination ratios with RFdiffusion. To ensure fairness, the length of each *de novo* sequence was set to 500 amino acids for all models. The entire evaluation process was conducted using a single GeForce RTX 4090 GPU. The hCAII (PDB: 1HEB) was fed into RFdiffusion with fixed regions to preserve the active site, while in ProteinMPNN, the number of generated sequences per structure was set to 100 for the generation of *de novo* carbonic anhydrase.

### Screening Platform and Structure Prediction for *De Novo* Enzymes

EC number prediction is using CLEAN, while protein sequences are prepared in TSV format, retrieved using ESM1b embedding, and processed using the split100 data set with the max-separation method for inference. For solubility prediction using SoDoPE, NetSolP, and GraphSol, protein sequences were formatted in FASTA, ensuring proper formatting and accuracy. Each prediction tool was executed with default settings to generate detailed reports containing solubility scores. In the case of GraphSol, feature maps—such as amino acid encoding, physicochemical properties, evolutionary information, and predicted structural properties—were generated using SPOT-Contact-LM.^50^ For protein structure prediction with trRosetta, the Yang-Server (https://yanglab.qd.sdu.edu.cn/trRosetta/) was used with the PDB template mode enabled.

### Molecular Dynamic Simulation and Molecular Docking

The three-dimensional (3D) structures were predicted by using trRosetta. The atomic coordinates of ligand CO_2_ were obtained from the protein data bank (PDB). CHARMM-GUI was used to construct the enzyme-ligand molecular model,^51^ where hydrogen atoms were added to the enzymes, and both enzymes and ligands were parametrized using the CHARMM36 force fields.^52^ The protein–ligand complex was then solvated using the TIP3P water model^53^ and ionized to simulate reaction conditions. The reaction was tested at 273 K with 0.05 M Zn^2+^. For the simulation of the dCAs/CO_2_ system, 0.15 M NaCl, 25 mM ZnSO_4_, and 25 mM Zn(OH)_2_ were added. Similarly, for the MD simulation of the dCAs/HCO_3_^–^ system, 0.15 M NaCl, 25 mM ZnSO_4_, and 25 mM Zn(HCO_3_)_2_ were included. The entire system was placed in a rectangular water box, with detailed dimensions provided in Table S1.

NAMD (version 3.0b3) was used for MD simulations with a 1 fs time step.^54^ The process began with 100,000 steps of energy minimization to resolve steric clashes, followed by 1 ns (1,000,000 steps) equilibration under the NVT ensemble and another 1 ns under the NPT ensemble. Temperature was maintained at 273 K *via* Langevin dynamics (damping coefficient: 1 ps^–1^) and pressure at 1 atm using the Langevin piston method. van der Waals interactions were truncated at 12 Å with a 10 Å^55^ switching function, and long-range electrostatics were calculated using the PME method with a 2 Å grid. Trajectories were analyzed by using VMD software. Long-range electrostatics were computed by using the PME method with a grid spacing of 2 Å. The trajectories were then analyzed for various properties using VMD software.^56^

### DNA Synthesis and Plasmid Construction

The DNA sequences of dCA12_2, dCA23_1, and hCAd4 were designed by using Integrated DNA Technologies (IDT) and optimized with TISigner. The optimized sequences were then submitted to IDT again to verify the synthesis validity. The protein and DNA sequence of dCA12_2 are provided in Table S2, dCA23_1 in Table S3, and hCAd4 in Table S4. After synthesis, the pET28a-placI-sfGFP vector was used for construct assembly. For the expression of hCAII, plasmid pET28a-hCAII was selected as the expression vector. Plasmid extraction was performed using the FAVORGEN extraction kit (Taiwan), and *Escherichia coli* BL21 (DE3) was used as the expression host *via* heat shock transformation. All strains, plasmids, and primers used in this study are listed in Tables S5 and S6.

### Culture Condition and Optical Density Measurement

Luria–Bertani (LB) medium (10 g/L tryptone, 10 g/L NaCl, and 5 g/L yeast extract) was used for culturing the recombinant strains. LB agar plates containing 50 μg/mL kanamycin were used to grow the strains. For the culturing process, a single colony was picked from the agar plate and inoculated into LB medium with 50 μg/mL kanamycin at 37 °C under 180 rpm agitation for 12 h as a preculture. Subsequently, 1% (v/v) of the preculture was inoculated into a 250 mL baffled flask containing 50 mL of LB medium with the same antibiotic and grown at 37 °C. When the optical density at 600 nm (OD_600_) reached 0.6, the culture was induced with 0.1 mM IPTG and incubated at 22 °C for 24 h to allow recombinant protein expression. Cell growth was monitored by measuring the OD_600_ with a spectrophotometer (Molecular Devices 384PC).

### SDS-PAGE Analysis

Sodium dodecyl sulfate polyacrylamide gel electrophoresis (SDS-PAGE) was used to analyze the protein expression. First, cells were harvested by centrifuging at 10,000*g* for 10 min. After discarding the supernatant, the cells were washed twice and then adjusted to an OD_600_ of 4 using deionized water with protein dye. A 12% SDS-PAGE gel was prepared to observe the protein expression. Finally, the proteins separated by SDS-PAGE were stained using Coomassie blue R-250, and the results were visualized by scanning with an Image Scanner (Biolab2000, Taiwan).

### Quantification of Protein Solubility via ImageJ

Protein solubility was quantified using ImageJ.^57^ The SDS-PAGE images were first converted to an 8-bit grayscale format. Individual bands corresponding to hCAII were selected by using a rectangular selection tool. The quantification of the target bands was then performed by measuring the peak intensity, where the peak height corresponded to the density of the protein bands.

### Carbonic Anhydrase Activity Characterization

Carbonic anhydrase (CA) activity was quantified using a modified Wilbur-Anderson (WAU) assay.^58^ The reaction system consisted of 9 mL of ice-cold Tris-HCl buffer (20 mM, pH 8.3) and 0.2 mL of enzyme sample, placed in a stirred 20 mL vessel. The reaction was initiated by the rapid addition of 6 mL of CO_2_-saturated ice-cold water. The time required for the pH to decrease from 8.3 to 6.6 was recorded. Enzyme activity was calculated using the formula: WAU = (*T*_0_ – *T*)/*T*, where *T*_0_ is the time taken for the pH change in the absence of enzyme (blank) and *T* is the time for the enzyme-containing samples.

## Data Availability

Data will be made available on reasonable request. The automatic script constructed in this study is available at https://github.com/Ryan-Hu-Hu-Hu/GRACE.

## Supporting Information Available

Compares amino acid frequency distributions from Progen2, EvoDiff, and DPLM with the Uniref50 database under different conditions (Figures S1); showed amino acid frequency distributions among all models (Figure S2); showed amino acid frequency distributions under various ratio of structures (Figures S3 and S4); Figure S5 presented protein expression, while Figure S6 illustrated the predicted structures of *de novo* designed enzymes dCA12_2 and dCA23_1, highlighting catalytic residues; showed the design concept and the experimental validation of hCAd4 (Figure S7); provided the detailed box size setting in the simulation systems (Table S1); provided information on engineered enzymes and DNA sequences, strains, plasmids, and cloning primers (Tables S2–S6) (PDF)
