# Supplementary material for: GRACE: Generative Redesign in Artificial Computational Enzymology
Source: ACS Synth Biol. 2024 Nov 8;13(12):4154–64. doi: 10.1021/acssynbio.4c00624 (PMC11669173; doi:10.1021/acssynbio.4c00624)
Supplement: Supplementary file 1 — sb4c00624_si_001.pdf [file sb4c00624_si_001.pdf]

Supporting information

GRACE: Generative Redesign in Artificial Computational Enzymology

Ruei-En Hu[1], Chi-Hua Yu[**,2], I-Son Ng[*,1]

[1]Department of Chemical Engineering, National Cheng Kung University, Tainan City 701, Taiwan

[2]Department of Engineering Science, National Cheng Kung University, Tainan City 701, Taiwan

*Corresponding author: Prof. I-Son Ng

Tel: +866-62757575; Fax: +886-62344496

E-mail: yswu@mail.ncku.edu.tw

ORCID: 0000-0003-1659-5814

**Co-Corresponding author: Prof. Chi-Hua Yu

E-mail: jonnyyu@gs.ncku.edu.tw

ORCID: 0000-0001-9445-3358

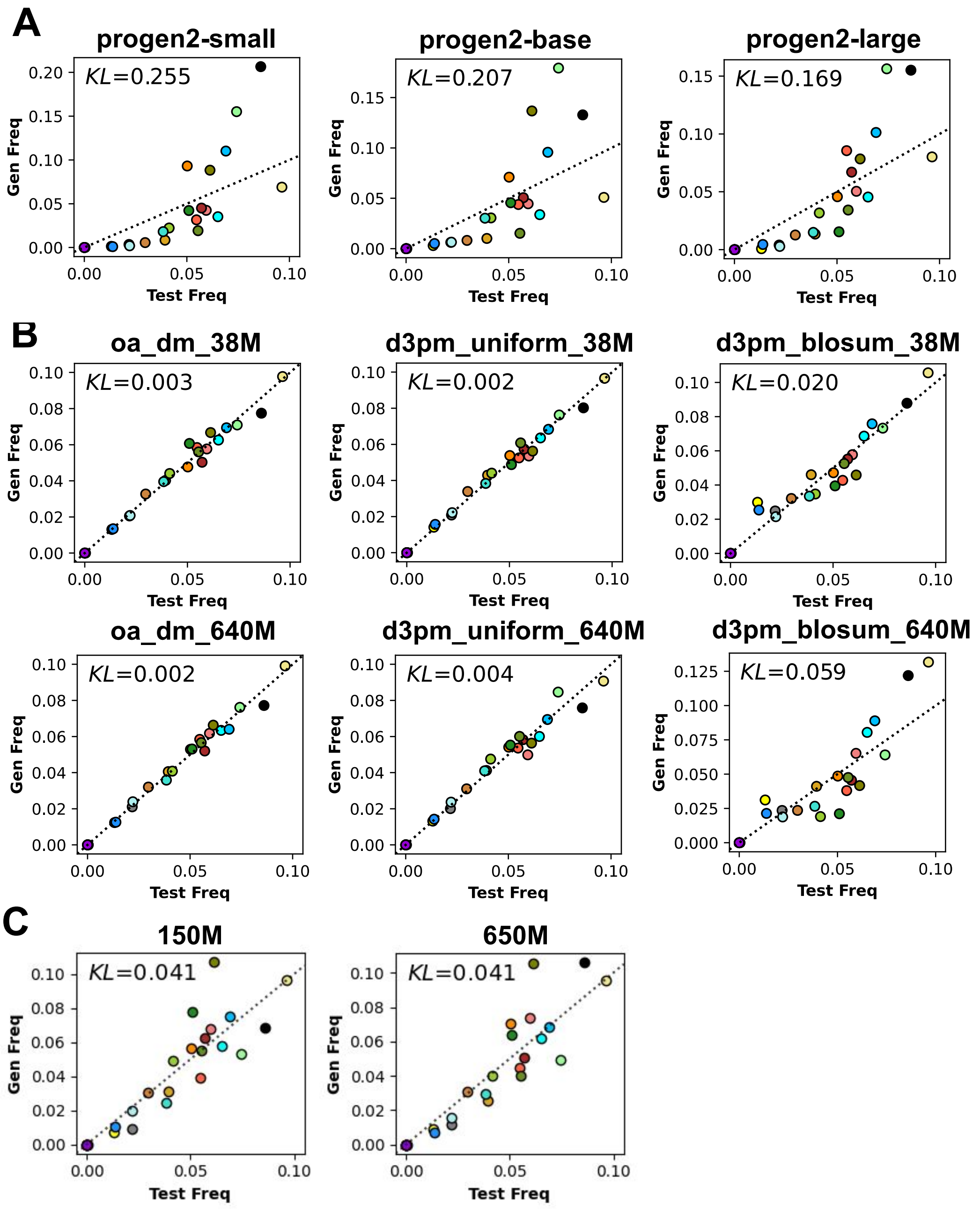

Figure S1. Amino acid frequency distribution of generated sequences (Gen Freq) plotted against the amino acid frequency distribution of the Uniref50 database (Test Freq). **(A)** Progen2 models, progen2-small, progen2-base, and progen2-large. **(B)** EvoDiff models, oa_dm_38M, oa_dm_640M, d3pm_blosum_38M, d3pm_blosum_640M, d3pm_uniform_38M, and d3pm_uniform_640M. **(C)** DPLM models, 150M and 650M

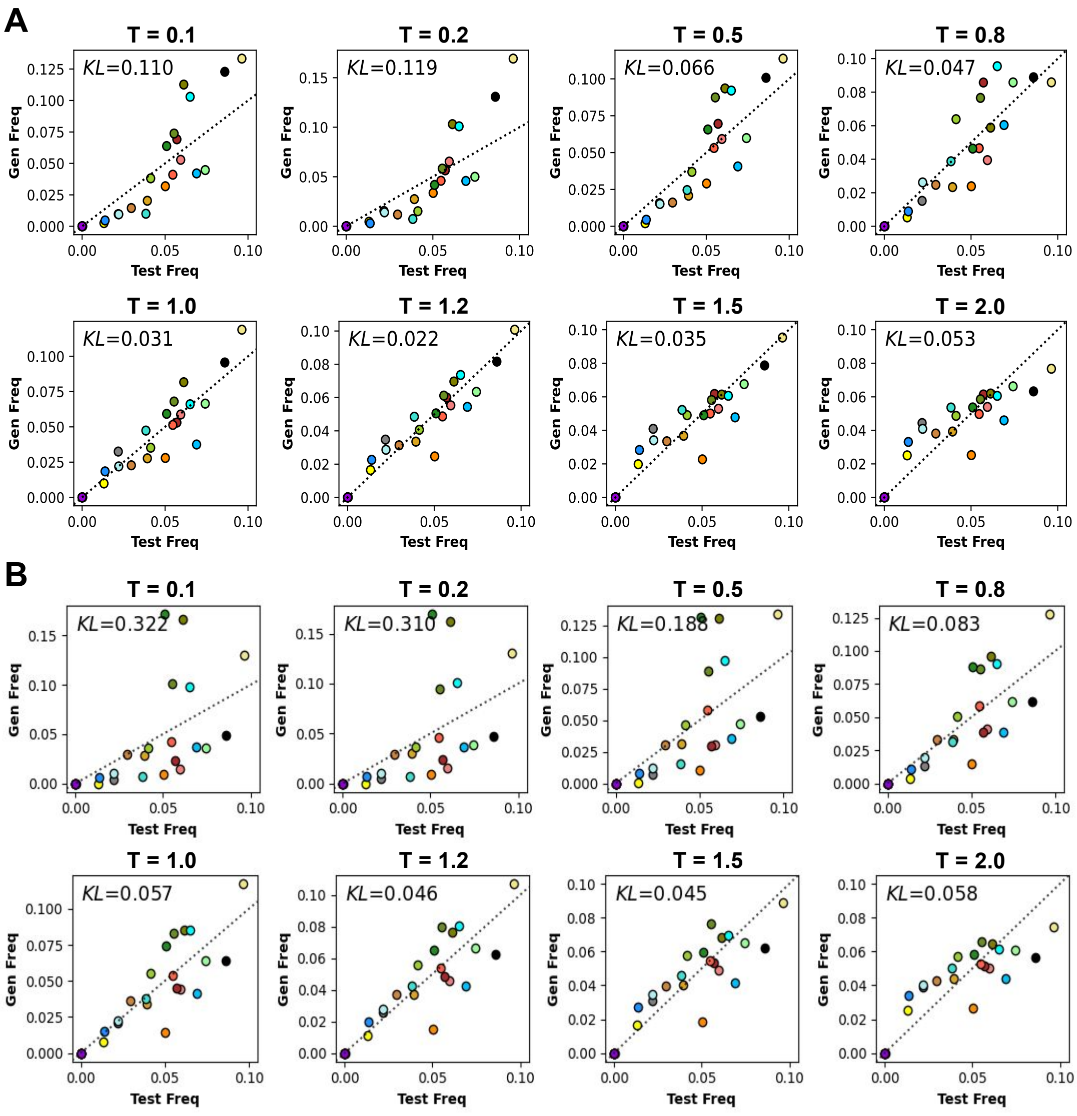

Figure S2. Amino acid frequency distribution of generated sequences (Gen Freq) plotted against the amino acid frequency distribution of the Uniref50 database (Test Freq). **(A)** RFdiffusion + ProteinMPNN using several sampling temperatures T at 0.1, 0.2, 0.5, 0.8, 1.0, 1.2, 1.5, and 2.0 with ratio of RFdiffusion to ProteinMPNN set at 1:100. **(B)** RFdiffusion + CarbonDesign using several sampling temperatures T at 0.1, 0.2, 0.5, 0.8, 1.0, 1.2, 1.5, and 2.0 with ratio of RFdiffusion to ProteinMPNN set at 1:100.

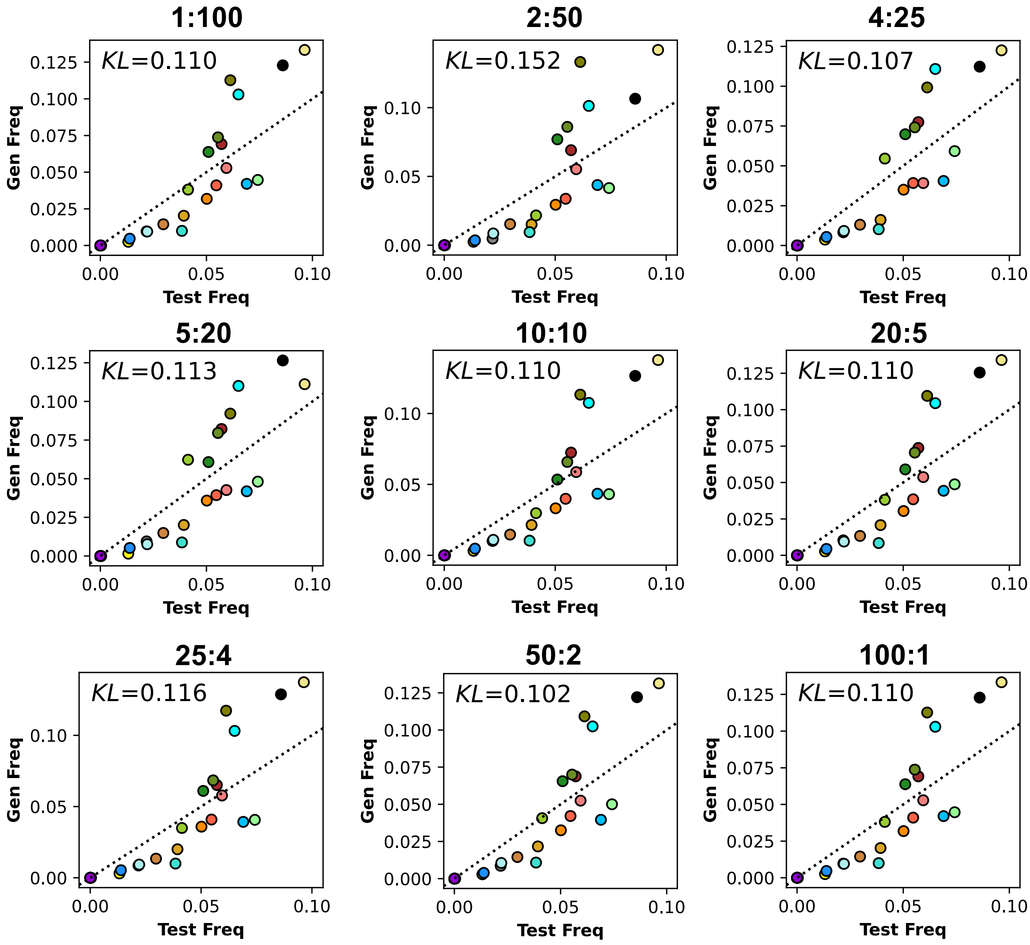

Figure S3. Amino acid frequency distribution of generated sequences (Gen Freq) at sampling temperature T = 0.1 plotted against the amino acid frequency distribution of the Uniref50 database (Test Freq). The notation "x:y" denotes the ratio of structures generated by RFdiffusion to sequences generated by ProteinMPNN per structure. Specifically, 1:100 indicates one structure generated by RFdiffusion with 100 corresponding sequences produced by ProteinMPNN; 2:50 signifies two structures generated by RFdiffusion, each yielding 50 sequences via ProteinMPNN, and so forth. This representation allows for the comparative analysis of amino acid compositional bias between the generated sequences and the reference database.

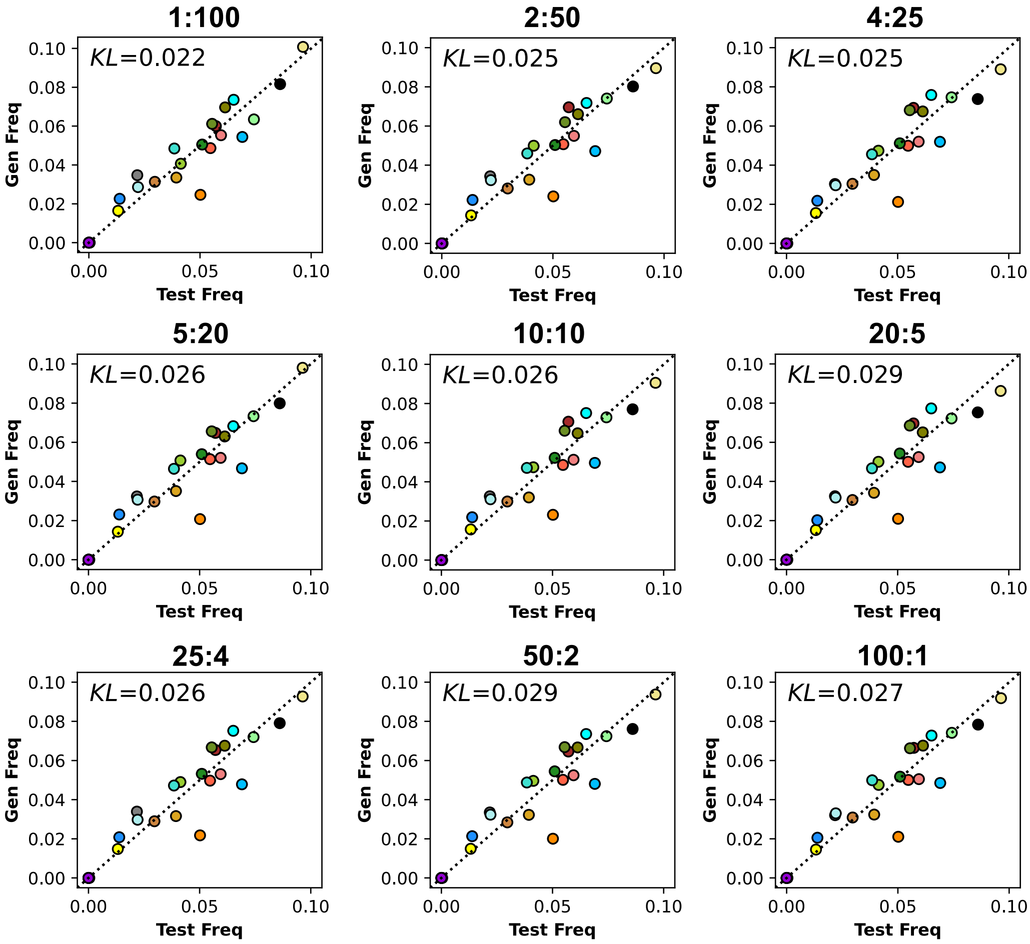

Figure S4. Amino acid frequency distribution of generated sequences (Gen Freq) at sampling temperature T = 1.2 plotted against the amino acid frequency distribution of the Uniref50 database (Test Freq). The notation "x:y" denotes the ratio of structures generated by RFdiffusion to sequences generated by ProteinMPNN per structure. Specifically, 1:100 indicates one structure generated by RFdiffusion with 100 corresponding sequences produced by ProteinMPNN; 2:50 signifies two structures generated by RFdiffusion, each yielding 50 sequences via ProteinMPNN, and so forth. This representation allows for the comparative analysis of amino acid compositional bias between the generated sequences and the reference database.

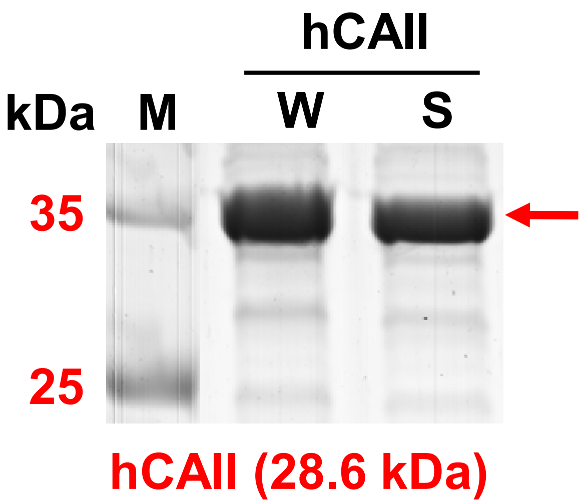

Figure S5. SDS-PAGE analysis of hCAII protein expression. W and S represent whole-cell and soluble proteins while the red arrow indicates the hCAII at 28.6 kDa.

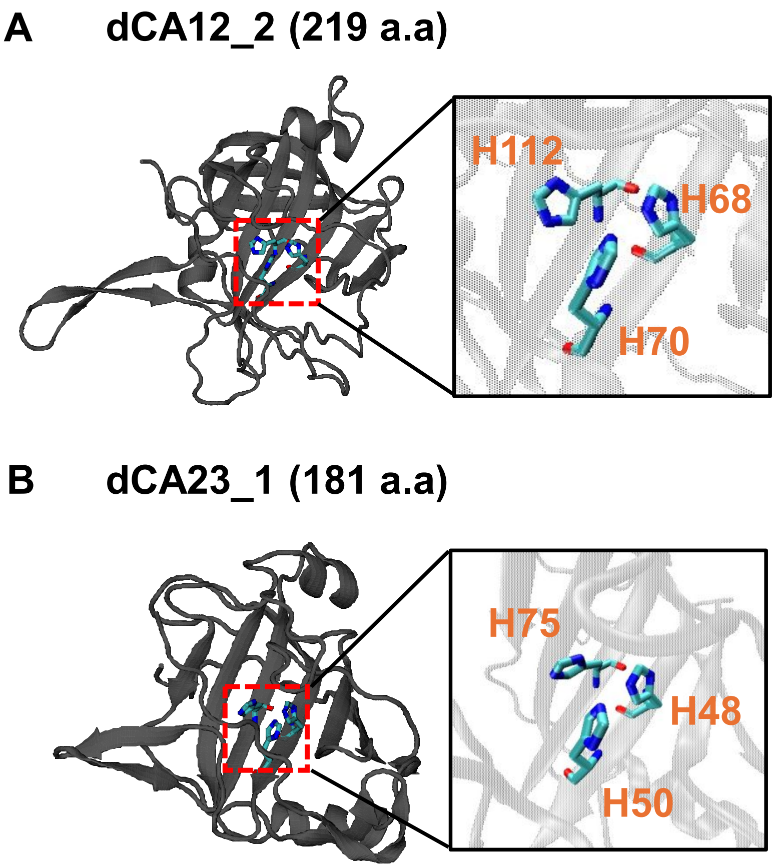

Figure S6. Predicted structure of de novo designed enzymes using trRosetta with corresponding catalytic triads highlighted. (**A**) dCA12_2 (219 amino acids) showing catalytic residues H68, H70, and H112. (**B**) dCA23_1 (181 amino acids) displaying catalytic residues H48, H50, and H75.

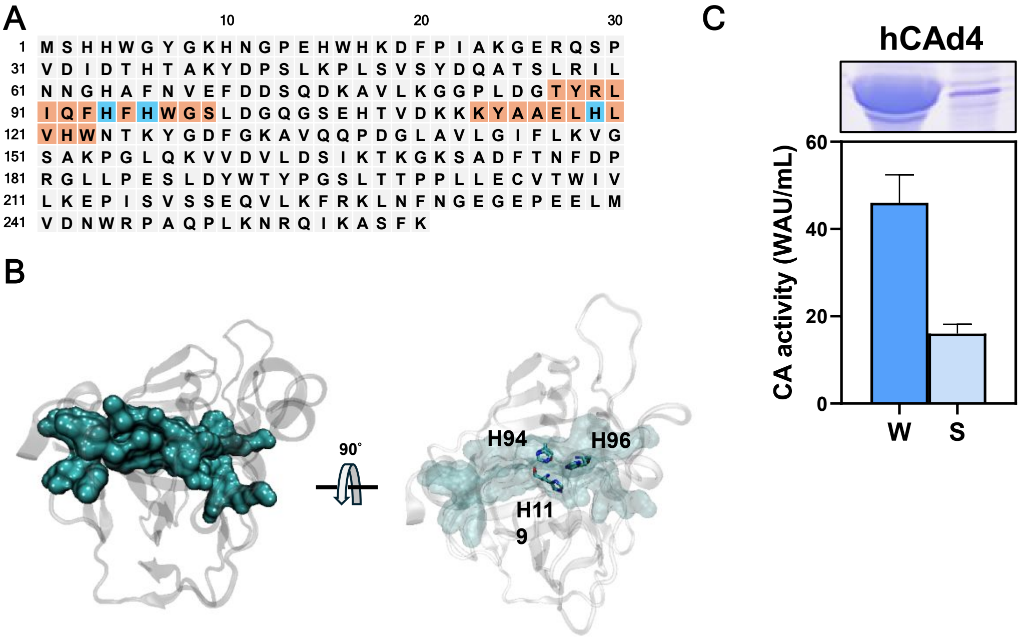

Figure S7. The motif recognition and selection of human carbonic anhydrase (hCAII) with enzyme characterization in hCAd4 design (A) Protein sequence of hCAII including the selected motifs are shown as orange color, and the catalytic triad H94, H96, and H119 are indicated by blue color. **(B)** The 3D structure of hCAII, in which the selected motifs (cyan color) form a deep cavity with catalytic triad H94, H96, and H119 laid at the bottom of the cavity. **(C)** The CA activity (bottom) and the corresponding protein expression (top) of hCAd4. W and S represent whole cell and soluble crude protein, respectively.

Table S1. The detailed box size information of each simulation system

System	Simulation box size (Å)
dCA12_2/CO_2	87 x 87 x 87 Å
dCA23_1/CO_2	78 x 78 x 78 Å
dCA12_2/HCO_3^-	86 x 86 x 86 Å
dCA23_1/HCO_3^-	74 x 74 x 74 Å

Table S2. The protein sequence and the decoded DNA sequence of dCA12_2

Protein sequence
MVVEEEVESWGYGGGILKYRLVDPEAKELRGLNNGHAFNVEFDDSTAPEV<br>ELVAEGRPLGRYRLIQFHFHWGSGLAVPGAEPTGSEHTETREVLAPDGTVK<br>TETVKYAAELHLVHWERSAEVTDFGKAVERGALAVLGIFLDLNSPETTVTV<br>EDVEKGKSWLESGGRAGRGSETTPPLLECVRWIVLPDVVREPAHVTLGRN<br>GGKLMVDNWRPRTVEEV
DNA sequence
ATGGTAGTCGAAGAAGAAGTCGAATCCTGGGGCTACGGGGGTGGAATC<br>TTAAAATATCGTTTAGTCGACCCGGAGGCGAAAGAGCTGCGTGGCTTAA<br>ATAACGGGCATGCCTTTAATGTCGAGTTCGACGATTCAACCGCACCTGA<br>AGTGGAGTTGGTGGCCGAAGGGCGTCCACTTGGTCGTTATCGCTTGATT<br>CAATTTCACTTTCATTGGGGCTCTGGGTTAGCTGTACCGGGAGCTGAGC<br>CGACCGGCTCCGAACATACGGAAACACGTGAGGTCTTAGCACCTGATGG<br>CACCGTCAAGACAGAGACCGTCAAGTATGCCGCTGAACTGCACCTTGTA<br>CACTGGGAGCGCAGTGCTGAAGTGACTGATTTCGGAAAAGCCGTGGAG<br>CGTGGAGCGCTGGCCGTACTGGGCATTTTTTTAGACTTAAATTCTCCCGA<br>GACTACTGTCACTGTAGAAGATGTGGAAAAAGGTAAGTCCTGGTTGGAA<br>TCTGGAGGTCGTGCTGGACGTGGGTCCGAGACTACTCCACCATTGTTGG<br>AGTGTGTACGTTGGATTGTACTGCCGGACGTAGTTCGTGAGCCAGCACA<br>CGTAACTCTGGGTCGCAACGGCGGAAAGTTGATGGTAGATAATTGGCGT<br>CCACGTACCGTCGAGGAGGTT

Table S3. The protein sequence and the decoded DNA sequence of dCA23_1

Protein sequence
MPTIKEWGYGRLRKKALNNGHAFNVEFDDSGDEAIKFPNGKLKRLIQFHFH<br>WGSTDGSEHTVVVNGKTIKYAAELHLVHWDKDLDFGKAVEKKNGLAVLG<br>IFLDKEYVEAMRSKLMEKGKSEGESLGGGSETTPPLLECVSPGEGWIVLKD<br>VLKGAKPLIHGSGRLPGKLMVDNWREYTVYL
DNA sequence
ATGCCAACTATAAAAGAATGGGGATACGGTCGTCTGCGTAAAAAGGCG<br>CTTAATAATGGTCACGCGTTTAACGTAGAGTTCGATGACAGTGGGGACG<br>AGGCCATCAAGTTTCCAAACGGAAAATTAAAGCGTTTAATCCAATTTCA<br>CTTTCATTGGGGATCTACCGACGGGTCCGAACACACCGTGGTCGTTAAT<br>GGGAAAACGATCAAGTATGCCGCAGAACTGCACTTAGTCCACTGGGAC<br>AAAGACTTAGATTTTGGTAAAGCCGTCGAGAAAAAAAATGGATTAGCA<br>GTTCTGGGAATTTTCTTAGACAAAGAGTATGTTGAGGCCATGCGCTCGA<br>AACTTATGGAGAAAGGCAAAAGTGAAGGGGAAAGCCTTGGCGGGGGAT<br>CTGAGACAACGCCGCCCCTGCTGGAGTGCGTGTCGCCCGGTGAGGGGTG<br>GATTGTGCTTAAAGACGTTCTGAAGGGCGCCAAGCCCCTTATTCACGGC<br>TCTGGGCGCTTGCCGGGCAAACTTATGGTAGATAACTGGCGTGAGTACA<br>CGGTCTACCTG

Table S4. The protein sequence and the decoded DNA sequence of hCAd4

Protein sequence
MEEYTIDLEPFKPLTLTLENGKTYRLIQFHFHWGSEVTEKGKYAAELHLVH<br>WDEEGNKHTITADIEFIGPCAGLFPKNLPEEVKKTSVETLRKNLEEALKQLG<br>ASPVEEKEEIIEPEGDSKKPPLKVKKIKVKLLKPITRADQVFLAVLLNIVEKK<br>RIEEELKDEVKEKPIFVNRDLLSEEAKKYRKEVLKEKGIEDVELEIQELIYER<br>TIKYAQENRPDSLLVIQEIELSDEYVKERMEEISSKENAEKYKEFLERFKKIIE<br>EAEKKGEKRFPKELEKASIEFLRETGRIGMFGARIPRE
DNA sequence
ATGGAGGAGTACACGATCGACTTGGAACCCTTTAAACCTTTAACGTTAA<br>CACTGGAGAACGGGAAGACTTATCGCTTGATTCAATTCCATTTTCATTG<br>GGGGTCAGAGGTTACGGAAAAGGGCAAATACGCTGCGGAACTTCATCT<br>GGTGCATTGGGACGAAGAGGGAAACAAGCATACTATTACCGCGGATAT<br>CGAATTCATCGGGCCATGCGCAGGGCTGTTTCCAAAAAATCTTCCCGAG<br>GAAGTTAAAAAAACTTCAGTCGAAACATTGCGTAAGAATTTGGAGGAA<br>GCCCTGAAGCAATTAGGTGCATCCCCAGTCGAGGAAAAGGAAGAGATC<br>ATTGAACCGGAGGGAGACTCGAAGAAGCCTCCGCTGAAGGTCAAAAAG<br>ATTAAAGTGAAGCTGTTAAAACCCATCACTCGTGCTGACCAAGTGTTCC<br>TGGCAGTCTTATTAAACATCGTCGAGAAGAAGCGCATTGAAGAAGAGTT<br>AAAGGACGAGGTAAAGGAGAAACCCATTTTCGTGAACCGCGATTTACT<br>GTCGGAAGAGGCGAAGAAATATCGTAAGGAGGTTTTGAAAGAGAAAGG<br>TATCGAAGACGTAGAGCTTGAAATTCAGGAGTTAATTTATGAACGCACA<br>ATTAAGTACGCCCAGGAGAATCGTCCAGACTCACTGCTGGTCATCCAGG<br>AAATCGAGCTGTCGGATGAATATGTGAAGGAGCGCATGGAGGAGATTT<br>CGAGTAAAGAAAATGCGGAGAAGTACAAAGAATTTTTGGAGCGTTTCA<br>AGAAGATCATCGAGGAAGCAGAAAAGAAGGGTGAGAAACGCTTTCCAA<br>AAGAACTTGAAAAAGCAAGTATTGAGTTCTTACGCGAGACGGGGCGCA<br>TCGGGATGTTCGGGGCTCGTATTCCGCGCGAGTGA

Table S5. Strains and plasmid used in the study.

Materials	Description	Remark
***E. coli* strains**		
DH5α	F-, Δ(argF-lac)169, ϕ80dlacZ58(M15), ΔphoA8, glnX44(AS), λ-, deoR481, rfbC1, gyrA96(NalR), recA1, endA1, thiE1, hsdR17	Genedirex
BL21(DE3) (BD in short)	F- ompT gal dcm lon hsdS$_B$(r$_B^-$m$_B^-$) λ(DE3 [lacI lacUV5-T7p07 ind1 sam7 nin5]) [malB$^+$]$_{K-12}$(λ^s)	Genedirex
**Plasmids**		
pET28a-placI-sfGFP	6423 bp, Km^R, pBR322 ori, placI driven *sfGFP*	Lab stock
pET28a-hCAII (hCAII in short)	6076 bp, Km^R, pBR322 ori, T7 driven *hCAII* with codon-optimized	Lab stock
pET28a-dCA12_2 (dCA12_2 in short)	5953 bp, Km^R, pBR322 ori, T7 driven *dCA12_2*	This study
pET28a-dCA23_1 (dCA23_1 in short)	5842 bp, Km^R, pBR322 ori, T7 driven *dCA23_1*	This study
pET28a-hCAd4 (hCAd4 in short)	6202 bp, Km^R, pBR322 ori, T7 driven *hCAd4*	This study

Table S6. Primer used in the study.

Primers	Description
NdeI-dCA12_2-F	GCCATATGGTAGTCGAAGAAGAAGTCGAATC
XhoI-dCA12_2-R	TGCTCGAGTCAAACCTCCTCGACGGTAC
NdeI-dCA23_1-F	GCCATATGCCAACTATAAAAGAATGGGGATAC
XhoI-dCA23_1-R	TGCTCGAGTCACAGGTAGACCGTGTACTC
NdeI-hCAd4-F	ATCATATGATGGAGGAGTACACGATCGA
XhoI-hCAd4-R	TGCTCGAGTCACGTCACTGCCCACGTCCCGATT

Restriction sites are indicated using underline.
